# Case report: Surgical treatment of McCune-Albright syndrome with hyperthyroidism and retrosternal goiter: A case report and literature review

**DOI:** 10.3389/fsurg.2022.921427

**Published:** 2023-01-06

**Authors:** Zhiwei Xing, Gongshuai Tao, Wanwan Pan, Delin Wu, Tingting Pan, Lingfeng Wan, Xiaopeng Ma, Yangyi Wang

**Affiliations:** ^1^ Graduate School, Wannan Medical College, Wuhu, China; ^2^ Department of Thyroid and Breast Surgery, The First Affiliated Hospital of USTC, Division of Life Sciences and Medicine, University of Science and Technology of China, Hefei, China

**Keywords:** retrosternal goiter, McCune Albright syndrome, hyperthyroidism, thyroidectomy, 131I treatment, case report

## Abstract

**Introduction:**

McCune-Albright syndrome (MAS) is a low-incidence syndrome consisting of the clinical triad of fibrous structural dysplasia of bone, endocrine disease, and skin pigmentation. Thyroid dysfunction is the second most common endocrine dysregulation in MAS. However, there are no treatment guidelines for MAS complicated with hyperthyroidism. Notably, no case of MAS complicated with retrosternal goiter and hyperthyroidism has been reported to our knowledge.

**Case presentation:**

We report a 27-year-old man with MAS who developed the typical triad of bone fibrous dysplasia, skin pigmentation and hyperthyroidism, complaining of recent fast-growing neck mass and difficulty in breathing. Hyperthyrodism was under control by Thiamazole, and computed tomography showed an enlarged thyroid extending retrosternally. We performed a total thyroidectomy on the patient. At the 1-year follow-up, the patient's dyspnea, hyperthyroidism, and bone pain were all significantly alleviated.

**Review:**

We searched the literature for previous case reports concerning MAS patients complicated with thyroid dysregulation. A total of 17 articles and 22 patients were identified to form our database. Among them, 9 studies clearly mentioned surgical intervention in 11 patients, and prognoses were also reported. Surgery was the most common intervention chosen and indicated a satisfactory prognosis.

**Conclusion:**

We report a rare case of MAS patient complicated with retrosternal goiter and hyperthyroidism. Our review provides an overview of MAS cases requiring interventions on thyroid function, and total thyroidectomy should be a proper treatment for these patients.

## Introduction

First reported in 1936, McCune-Albright syndrome (MAS) is a rare congenital sporadic disorder with an estimated prevalence ranging from 1 in 100,000 to 1 in 1,000,000 (1–3). It consists of a clinical triad including polyostotic fibrous dysplasia, skin pigmentation, and hyperfunctional endocrine diseases. Increased hormone production leads to various endocrine diseases, such as precocious puberty, hyperthyroidism, excess growth hormone, and Cushing syndrome. Among them, hyperthyroidism is the second most common endocrine dysregulation.

The main treatment for MAS complicated by hyperthyroidism includes drug therapy, ^131^I radiotherapy, and surgical resection. Due to the sparseness of cases, no studies have compared the effect of each treatment method to our knowledge. Moreover, retrosternal goiter has not been reported in MAS, making the evidence-based management of patients even more challenging. In this study, we report a 27-year-old male patient diagnosed with MAS complicated by retrosternal goiter at our institution. Through literature research, we aim to collect MAS cases complicated with hyperthyroidism in which drug therapy is not sufficient and surgical intervention on thyroid may play a role, and analyze the clinical features, treatments, and outcomes of these patients.

## Case presentation

**Figure 1:**
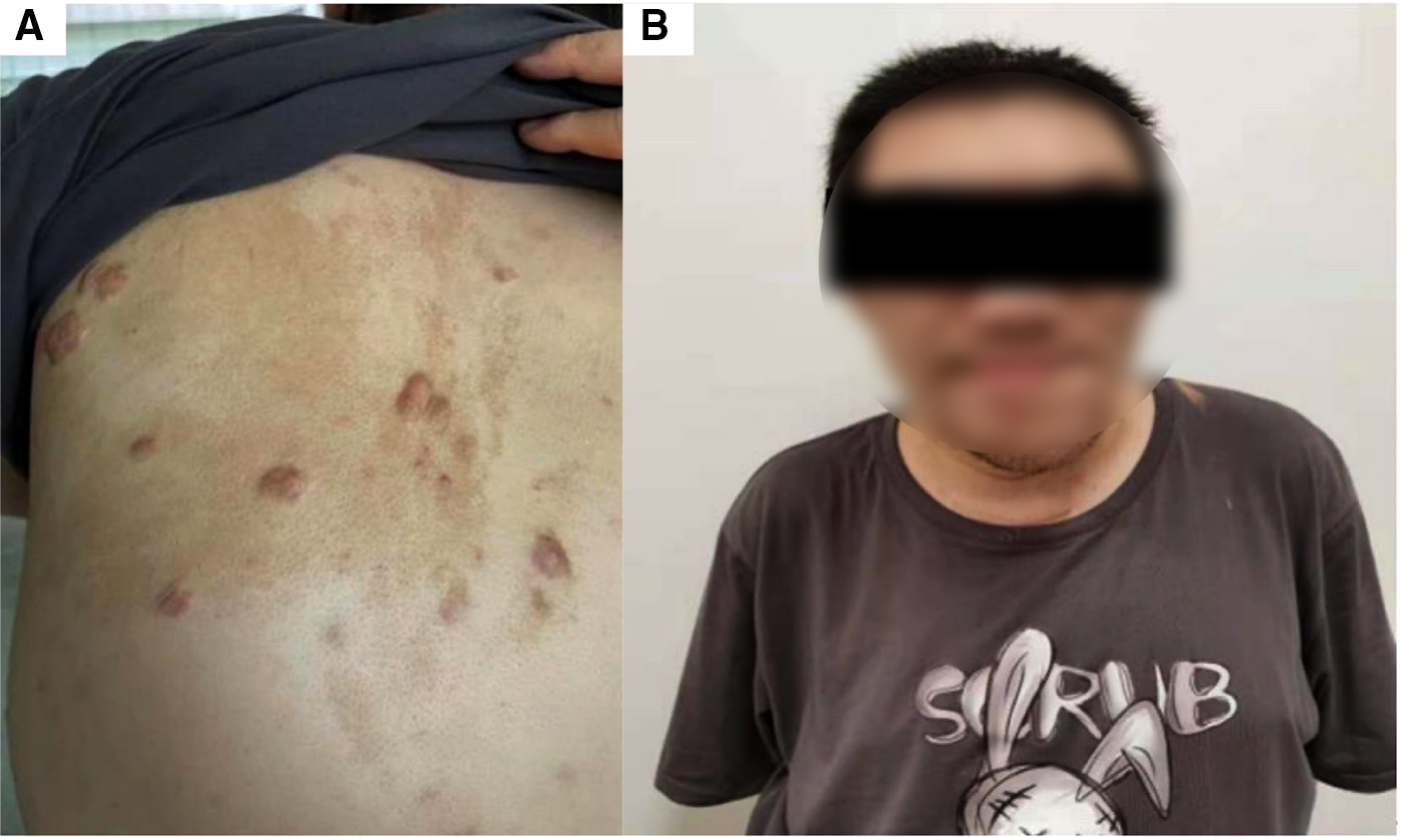
Representative images of the patient indicating McCune-Albright syndrome. Photographs of the back (**A**) from this patient demonstrates characteristic light brown pigmented birthmarks with jagged borders. These flat patches occur around the midline of the back and are known as Café-au-lait spots. The appearance of the patient's face (**B**) is indicative of a fibrous dysplasia disorder.

The patient, a now 28-year-old male, was born with café-au–lait macules on the left side of the back (Figure 1A). He was initially symptomatic with a slight facial deformity and blurred vision in 2008. In 2009, he was diagnosed with bone fibrous dysplasia and treated with Calcium Carbonate 1,200 mg once daily (QD), Vitamin D_3_ Tablets 600 mg QD and calcitriol 0.25 μg QD for 2 months and disodium chlorophosphite 1,600 mg QD for 2 years. In 2014, his facial deformities worsened, most notably in the left mandible, while he presented with more symptoms including widened teeth, wide lips, enlarged nose, occasional palpitations, and sleep snoring (Figure 1B). In 2015, he underwent left mandibular abrasion; since a left humeral fracture occurred out of minimal trauma injury during hospitalization, an external stent fixation was performed. Zoledronic acid 5 mg was injected yearly to relieve bone pain.

Also in 2015 during hospitation, due to palpitations, thyroid function was tested. With a Free Triiodothyronine level of 11.46 pg/ml, a free thyroxine level of 3.36 ng/dl, and a thyroid-stimulating hormone (TSH) level of <0.008 μIU/ml, a diagnosis of hyperthyroidism was made. Thiamazole 5 mg twice daily was prescribed since then. In 2018, during annual health check-up, the patient was diagnosed with bilateral thyroid nodules for the first time by Doppler ultrasound (data unavailable) and left untreated. In 2019, thyroid ultrasound follow-up indicated the nodules to be 7.2 cm × 6.7 cm × 4.4 cm on the left and 1.9 cm × 1.7 cm × 1.0 cm on the right. In 2020, the size increased to 8.3 cm × 5.6 cm × 4.7 cm on the left and 2.7 cm × 2.0 cm × 1.0 cm on the right.

**Figure 2:**
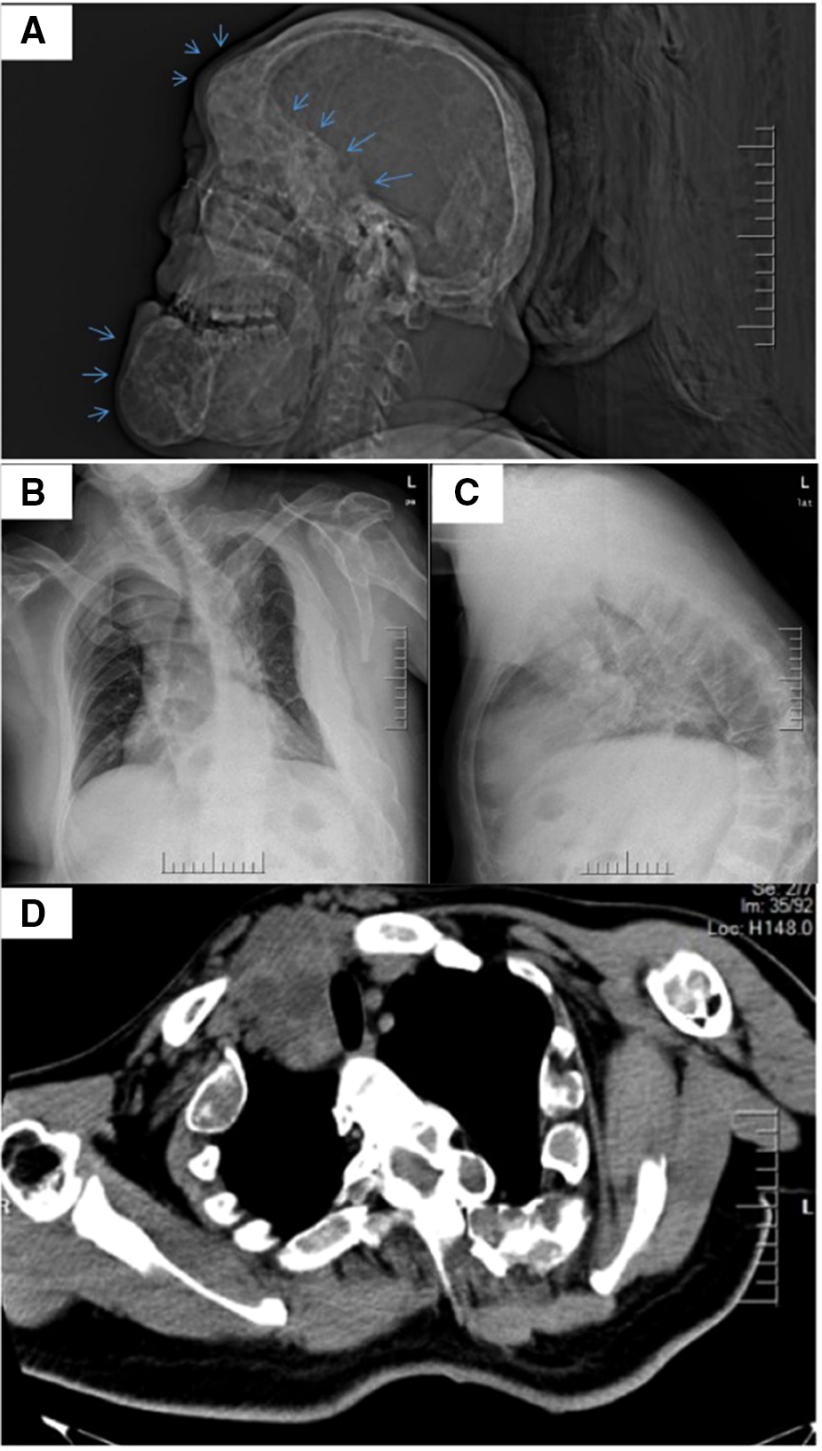
Radiographic features of the patient before surgery demonstrating fibrous dysplasia and an enlarged thyroid. The lateral skull radiograph acquired from a CT (**A**) indicates polyostotic fibrous dysplasia on the head (blue arrows). X-ray from the patient (**B,C**) shows thoraco-lumbar scoliosis. CT image of patient upper chest (**D**) demonstrates the enlarged retrosternal goiter.

In 2021, the patient presented to the thyroid clinic complaining of chest tightness and shortness of breath after minimal activity. The patient denied a history of oncological diseases, other genetic diseases, exposure to epidemic areas, special chemicals, or radioactive materials. Upon physical examination, he was calm with a pulse rate of 78 beats/min and blood pressure of 135/78 mmHg. He had firm, nontender swelling on both lobes of the thyroid, 9.0 cm × 6.0 cm on the left and 6.0 cm × 4.0 cm on the right. The trachea deviated to the left. Mixed echo nodules were identified in both lobes of the thyroid gland (Ti-RADS grade, level 3) on Doppler ultrasound. Computed tomograpy (CT) showed heterogeneously decreased bone density in the skull, face, and skull base, showing “diffuse ground glass” changes. Polyostotic fibrous dysplasia was prominent on CT with the presence of a thickened skull plate, asymmetrical facial bone, and the osteolytic changes of the mandible (Figure 2A). A normal hemogram and serum biochemistry were obtained, and thyroid function was also within a normal range, with triiodothyronine of 2.13 nmol/L, thyroxine of 117.03 nmol/L, and TSH of 0.945 mIU/L. Further investigations showed thyroglobulin antibody of 0.00 IU/ml (N, 0–4.91), and thyroid peroxidase antibody of 0.50 IU/ml (N, 0–9), while the patient's thyroglobulin level increased to 487.00 μg/L (N, 1.15–130.77). Cardiac ultrasound pointed out whole heart enlargement, with an ejection fraction of 65% (normal range: 50%–70%). Chest radiograph revealed scoliosis and kyphosis, together with a thoracic deformity (Figures 2B,C). Chest CT scan showed a right thyroid goiter (62 mm × 72 mm) around the trachea. Due to the compression from the goiter, the diameter of the trachea was approximately 18 mm at the narrowest point (Figure 2D). The preoperative diagnosis was (1) bilateral thyroid nodules; (2) retrosternal goiter; (3) hyperthyroidism and (4) polyostotic fibrous dysplasia.

**Figure 3:**
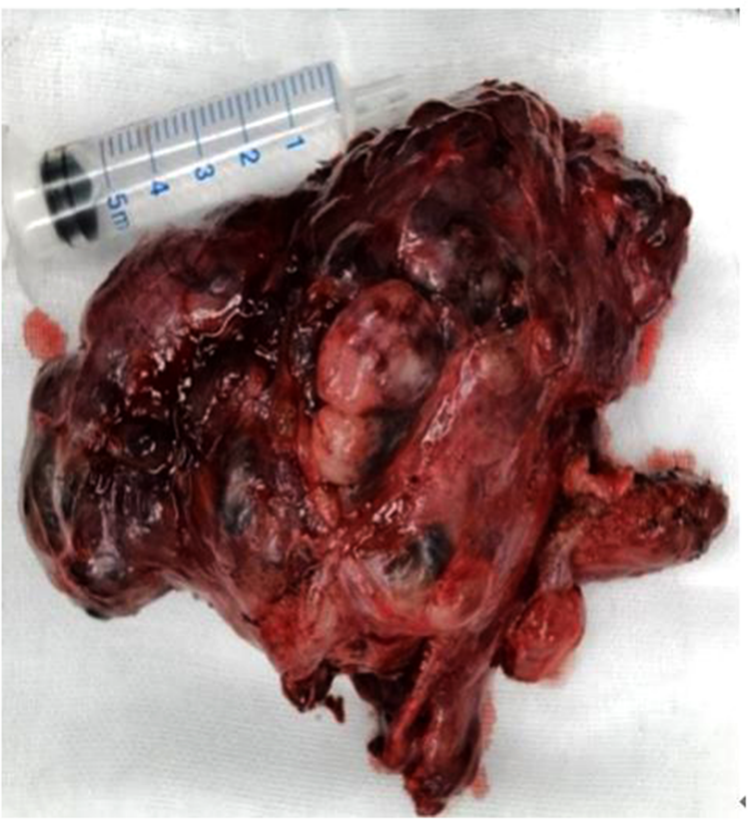
Resected thyroid gland from the patient.

**Figure 4:**
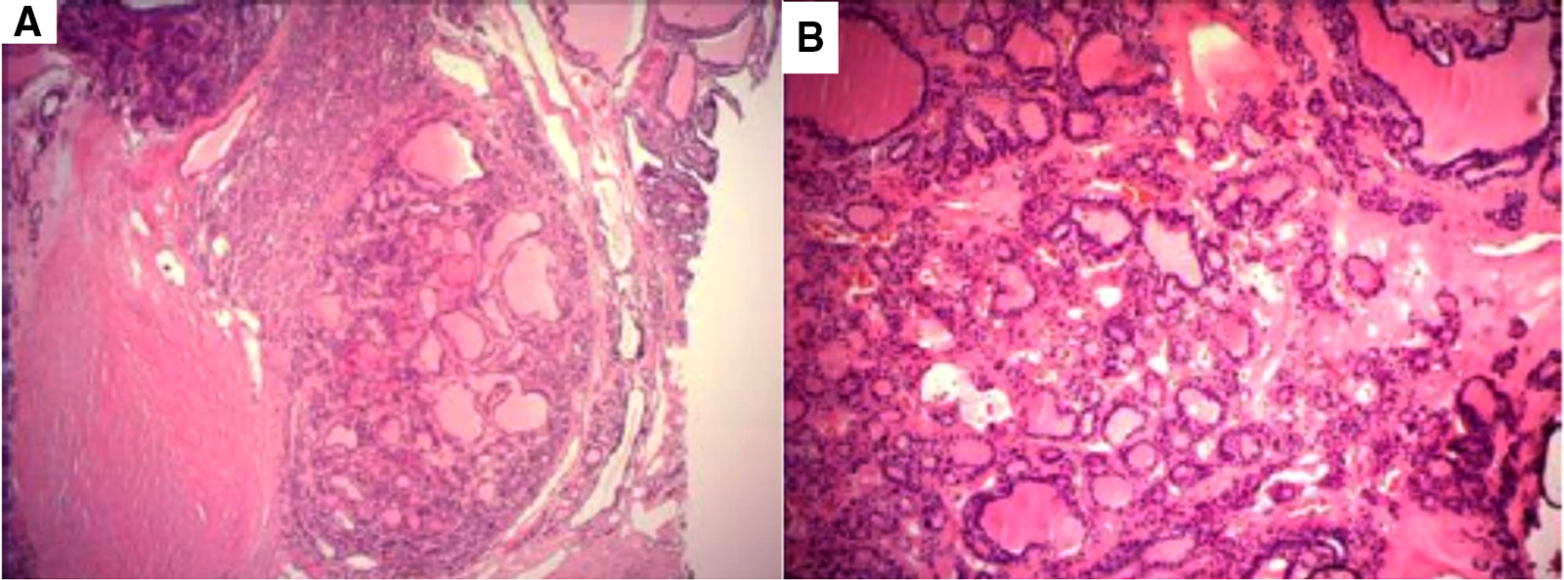
Histopathological images of the resected thyroid specimen. (**A**) Adenomatous nodular goiter with cystic changes (magnification 40×). (**B**) Some thyroid follicular epithelial cells with papillary hyperplasia (magnification 100×).

Under general anesthesia with endotracheal intubation, a total thyroidectomy was performed. The blood supply of the significant goiter behind the sternum was extremely abundant, and the intraoperative bleeding was approximately 600 ml in total. This may result from the severe thoracic deformity which created ample room for the growth of the gland. Blood vessels on the thyroid surface were ligated as the first step to reduce bleeding. The parathyroid glands and the recurrent laryngeal nerves were difficult to locate, and intraoperative nerve monitoring was used to identify and preserve the nerves. Complete removal of the thyroid gland was performed and submitted for histopathological examination. Grossly, the surface of the specimen was pinky-grey and sized at 11 cm × 7 cm × 5 cm. Cut surface reveled asymmetrical multinodular changes, the largest being 2.5 cm in diameter (Figure 3). In H&E-stained paraffin sections, variably-sized dilated follicles showed nodular hyperplasia, with some uniform-sized follicles showing adenomatous hyperplasia (Figures 4A,B). Cystically-dilated follicles could be observed locally and contain large amount of colloid. Part of the follicular epithelium shows papillary hyperplasia (grade 1–2 papillae), round cells, oval cells, mild atypia, no clear nuclear grooves and intranuclear pseudo inclusions. Based on the naked eye and microscopic view, we confirmed the diagnosis of multinodular goiter. The patient was sent to the intensive care unit postoperatively and spontaneous respiration restored the next day.

Breathing difficulty was instantly relieved after surgery. The patient's postoperative course was uneventful. L-thyroxine was taken daily after surgery and the dosage was adjusted to assure a serum thyroxine level within the normal range and close to the lower limit. No residual thyroid gland or abnormal lymph nodes were detected in the postoperative ultrasound follow-up. One year after surgery, the patient stated that tolerance to activity had substantially improved. Also, bone pain in the lower limbs and back had alleviated, and no new fractures had happened.

## Literature review

Hyperthyroidism of this patient was initially controlled with thiamazole. The necessity of thyroidectomy originated from retrosternal goiter and limited physical capacity. However, it is not clear whether the long-term drug control of hyperthyroidism was proper, or should it be replaced earlier with more radical treatment like thyroidectomy and radioactive iodine (4). We searched the PubMed database using “McCune Albright syndrome and hyperthyroidism”, “McCune Albright syndrome and thyroidectomy”, and “McCune Albright syndrome and goiter” as keywords. The purpose of our literature research is to collect MAS cases complicated with thyroid dysregulation as many as possible in order to assess the clinical information, especially treatment and prognosis.

We collected a total of 17 articles that included 22 cases of MAS complicated with thyroid dysregulation. The patient clinical characteristics, thyroid dysregulation type, treatment and prognosis were listed in Table 1. Among them, 9 studies with 11 patients clearly mentioned surgery or ^131^I treatment. Treatment options and corresponding prognoses were summarized in Table 2.

**Table 1:** Characteristics, treatments, and prognoses of reported MAS with thyroid dysregulation.

No.	Publication time	Study/First Author	Sex/Age, years	Thyroid abnormality	First treatment	Prognosis
1	1951	Yettra (5)	F/40	/	Subtotal thyroidectomy	Three years later, the right hip was fractured, and 15 years later, the left mandible was enlarged, with two small nodules visible under the scar of the neck incision.
2	1973	Hamilton (6)	M/11	Hyperthyroidism	Subtotal thyroidectomy^131^I	One year later, the hyperthyroidism relapsed, and the patient continued medication until the age of 20. At the age of 35, she still had slowly progressive hyperthyroidism and facial bone deformities.
3	1974	Andrews (7)	F/17	Diffuse goiter Hyperthyroidism	Surgery (No excision range)	Pregnant was normal at age 19 and had no new fractures for the next 2 years.
4	1979	Richten (8)	F/4	Hyperthyroidism Nodular goiter	Subtotal thyroidectomy	The symptoms of the patient continued, the thyroid gland was abnormal at the age of 8 years old, and the facial bone deformity appeared.
5	1984	Hall (9)	M/22	Goiter	Subtotal thyroidectomy	Seven years later, the body shape began to change slightly, and 12 years later, the remaining thyroid (right side) began to increase, and symptoms gradually began to appear such as headache, enlarged left face and poor teeth.
6	1986	Lee (10)	F/10	Diffuse goiter Hyperthyroidism	Subtotal thyroidectomy	A partial thyrotomy was performed again at age 18, following three I treatments due to a recurrence of hyperthyroidism.
7	1986	Lee (10)	M/10	Hyperthyroidism	Subtotal thyroidectomy	He then received ^131^I at age 20 and 37.
8	1986	Lee (10)	F/13	Hyperthyroidism	^131^I	/
9	1992	Schwinding (11)	M/34	Hyperthyroidism	^131^I	There is a recurrence of hyperthyroidism and the occurrence of pathological fractures.
10	1997	Mastorakos (12)	F/6	Hyperthyroidism	^131^I	/
11	2003	Hannon (13)	M/14	Hyperthyroidism	Total thyroidectomy	/
12	2003	Collins (14)	F/14	Hyperthyroidism Papillary thyroid carcinoma	Subtotal thyroidectomy	Postoperative pathological diagnosis was thyroid papillary cancer, followed by total thyroidectomy, with no clinical symptoms within 40 months.
13	2003	Collins (14)	F/40	Thyroid cancer	Subtotal thyroidectomy	Total thyroidectomy was performed 4 months after surgery, and the patient had no other clinical symptoms for 51 months.
14	2007	Bhat (15)	F/30	Hyperthyroidism	Subtotal thyroidectomy	After surgery, the patient was treated with sodium palmirate, and no new fracture was found during 3 years of follow-up.
15	2011	Elhaï (16)	F/38	Hyperthyroidism Nodular goiter	Surgery (No excision range)	Postoperative bone pain was still present.
16	2012	Chakraborty (17)	F/7	Hyperthyroidism Diffuse goiter	^131^I	/
17	2013	Kollerova (18)	F/31	Hyperthyroidism Nodular goiter	Total thyroidectomy	Pathologic fractures occurred early after surgery, but no subsequent fractures occurred.
18	2018	Nakao (19)	M/31	Hyperthyroidism Goiter	Total thyroidectomy	/
19	2019	Merchant (20)	F/0.42	Hyperthyroidism	Total thyroidectomy	Two months after surgery, the ovarian cysts shrank, vaginal bleeding disappeared, and the patient's overall clinical condition improved thereafter.
20	2019	Merchant (20)	F/2	Hyperthyroidism Diffuse goiter	Total thyroidectomy	/
21	2019	Merchant (20)	F/4	Hyperthyroidism Diffuse goiter	Total thyroidectomy	/
22	2020	Legrand (21)	F/18	Hyperthyroidism Papillary thyroid carcinoma	Subtotal thyroidectomy	/

**Table 2:** Surgical and ^131^I interventions of thyroid in MAS patients.

Treatment	Number of cases	Patient serial number	Prognosis
Subtotal thyroidectomy	6 cases	1, 2, 4, 5, 6, 7	Relapse
^131^I	1 case	9	Relapse
Subtotal thyroidectomy and then total thyroidectomy	2 cases	12, 13	Satisfactory
Total thyroidectomy	2 cases	17, 19	Satisfactory

Among these 11 cases, 6 patients were reported to undergo partial thyroid resection, but the prognoses were poor mainly due to postoperative hyperthyroidism recurrence. Additionally, bone fibrosis tends to progress in these patients. One patient received solely ^131^I treatment, but the hyperthyroidism also recurred after treatment, and pathological fractures occurred. Two patients underwent partial thyroidectomies followed by total thyroidectomies, and the prognoses were reported to be relatively satisfactory. Only two patients underwent instant total thyroidectomies, and there were no recurrences or aggravation of hyperthyroidism.

Thyroid cancer was occasionally diagnosed in these MAS cases. Patients No. 12 and No. 22 underwent thyroidectomies for hyperthyroidism, but thyroid cancer was found in the postoperative pathological examination. Patient 13 underwent a fine-needle aspiration biopsy for thyroid nodule, suspicious cancer cells were identified and the diagnosis of thyroid cancer was confirmed with postoperative pathology.

## Discussion

MAS was initially defined with the typical clinical triad. The definition was further broadened in a 2004 literature based on 113 patients with MAS. Among these patients (98 females and 15 males), 24% had the typical triad, 33% had two of the symptoms, and 40% had only one. Despite diverse manifestations, it is now confirmed that MAS is caused by a somatic gene mutation of the GNAS gene encoded by the guanine-nucleotide-binding protein α-subunit in the early embryonic stage (22). The mutated GNAS gene leads to the activation of the TSH/G protein pathway and the subsequent elevated intracellular adenosine 3’,5’-cyclic monophosphate level (23, 24). Interestingly, for patients who had the typical triad, mutations of the GNAS gene were detected in 46% of the peripheral blood samples of the patients, while in patients with two or one symptom, this number dropped to 21% and 8%, respectively (25). As for the application of Polymerase Chain Reaction test on GNAS mutation, it is considered necessary only if the diagnosis remains uncertain after a physical examination, hormonal level evaluation, and radiologic evaluation of the skeletal (26). In a word, a typical clinical presentation is essential for the diagnosis of MAS rather than genetic testing on the affected tissue (bone, thyroid etc.) or peripheral blood (26). For our patient, genetic testing was unfortunately not performed due to financial reasons and clinical considerations, but the diagnosis of MAS remains validated.

It is well-accepted that thyroid dysfunction is common in MAS. Approximately 2/3 of patients with MAS will develop thyroid enlargement of varying degrees (27). Thyroid abnormalities are the second most common endocrine disease found in MAS, mostly hyperthyroidism and noticed between 14 and 15 years of age. The ratio of males to females is 1:3. P P. Feuillan and his colleagues analyzed the clinical data of 19 MAS patients and found that thyroid insufficiency is common, often clinically insidious and progresses slowly (27).

Thyroid dysregulation in MAS relates closely to bone fibrous dysplasia. Excessive thyroid hormone leads to a direct resorption of bone, so an early diagnosis and treatment of hyperthyroidism is important in MAS patients (27–29). A study based on 9 pediatric MAS patients showed that a major portion of pediatric patients were admitted to hospital due to fibrous bone dysplasia and it's possible to be exposed to the risk of developing endocrine diseases (13). Therefore, screening for associated risks is necessary for these patients (13). In our case, the thyroid disease progression may begin much earlier than the initial drug treatment, and the failure of early intervention also negatively impacted the progression of fibrous bone dysplasia.

The main treatment for MAS complicated by hyperthyroidism includes antithyroid drugs, ^131^I radiotherapy, and surgical resection. Although hyperthyroidism in MAS usually responds well to drug therapy, a complete cure is unlikely. More radical treatment includes thyroidectomy and ^131^I treatment. The mechanism of ^131^I treatment is through the intake of ^131^I by the thyroid gland and the release of β-rays. However, similar to the mechanism of partial thyroidectomy, ^131^I cannot destroy all normal thyroid tissue. The remnant thyroid tissue may continue to develop symptoms due to intrinsic gene mutations, as had happened to patients No.13.

Three patients were diagnosed with thyroid cancer, which is not rare considering our sample size. After close examination of the specimens their samples, mutations in the GNAS gene were found in thyroid cancer tissue, but not in the surrounding normal thyroid tissue in patients 12 and 13. We speculate that mutations in the GNAS gene are closely related to the occurrence of thyroid cancer. The possibility of thyroid cancer development should be alerted during the follow-up for MAS patients.

Regarding the treatment of hyperthyroidism in our patient, he had been taking thiamazole since the detection of hyperthyroidism. However, goiter still developed dramatically and became the first ever reported retrosternal goiter in MAS to our knowledge. Considering that this patient had severe scoliosis of the spine, the right thoracic space became larger, creating space behind the sternum for the enlarged thyroid gland to protrude, resulting in compression of the airway and symptoms of dyspnea. Based on the patient's clinical presentation, we chose to perform a total thyroidectomy. The difficulty of the operation in this patient was as follows: (1) A short neck, obesity with a body-mass index of 34.7, a round back, neck extension limitations, an abnormal facial structure, and tracheal deflection, and tracheal compression all increased the difficulty of intubation for anesthesia. (2) At the same time, due to severe thoracic deformity and size of the goiter, the lower edge of the lesion located behind the sternum. (3) The lesion was rich in blood supply and surgery through Kocher's incision was difficult. (4) Due to hyperplasia of the bone tissues, the patient had a predisposition towards spontaneous fractures. Nevertheless, the symptoms of dyspnea, snoring, hyperthyroidism, and lower limb and backbone pain all significantly alleviated during the postoperative follow-up. Surgical removal of the thyroid gland is a proper treatment for this patient, which may aid other patients in treatment selection.

In comparison, thyroidectomy has become the least commonly selected treatment for newly diagnosed Graves’ hyperthyroidism (30). For patients with MAS complicated by hyperthyroidism, due to insufficient samples, we cannot determine whether total thyroidectomy is the best approach, but from the analysis of existing cases, we conclude that (1) thyroid dysfunction is likely to reoccur after the cease of the antithyroid drug; (2) ^131^I radiotherapy and subtotal thyroidectomy may possibly lead to a recurrence of hyperthyroidism; and (3) total thyroidectomy not only cures hyperthyroidism but also suppresses the progression of bone fibrosis. Therefore, we are more inclined to choose total thyroidectomy in these patients rather than subtotal thyroidectomy or ^131^I radiotherapy.

## Data Availability

The original contributions presented in the study are included in the article/Supplementary Material, further inquiries can be directed to the corresponding authors.
